# Improving Indoor WiFi Localization by Using Machine Learning Techniques

**DOI:** 10.3390/s24196293

**Published:** 2024-09-28

**Authors:** Hanieh Esmaeili Gorjan, Víctor P. Gil Jiménez

**Affiliations:** Department of Signal Theory and Communications, Universidad Carlos III de Madrid, Av. de la Universidad, 30, Leganés, 28911 Madrid, Spain; 100446982@alumnos.uc3m.es

**Keywords:** WiFi positioning, machine learning, random forest, KNN, NN, catBoost, XGBoost, GridSearchCV

## Abstract

Accurate and robust positioning has become increasingly essential for emerging applications and services. While GPS (global positioning system) is widely used for outdoor environments, indoor positioning remains a challenging task. This paper presents a novel architecture for indoor positioning, leveraging machine learning techniques and a divide-and-conquer strategy to achieve low error estimates. The proposed method achieves an MAE (mean absolute error) of approximately 1 m for latitude and longitude. Our approach provides a precise and practical solution for indoor positioning. Additionally, some insights on the best machine learning techniques for these tasks are also envisaged.

## 1. Introduction

Nowadays, positioning is very important for most of the services provided to users for many reasons. For example, there are applications and services that need the exact position of the user to provide the service, such as navigation, emergency, user delivery, or even gaming. Additionally, with the explosion of IoT (internet of things) [1] and smart cities, the need for positioning has grown dramatically [2]. Moreover, if we add healthcare into the picture, the requirement of an accurate and robust localization becomes a must for analyzing alerts to be prepared or for preventing any insecure issues [3]. In outdoor scenarios, localization and tracking are usually carried out by some sort of GPS (global positioning system) [4]. However, for indoor scenarios, where the GPS is not available, or mixed scenarios—indoor and near indoor scenarios with low satellite coverage—the positioning has to be acquired in a different way. Those methods can be classified into four main groups, namely, the following: (a) Inertial systems that make use of sensors measuring user motion in order to estimate the relative changes in their position; (b) *Radio frequency systems*, using technologies that transmit/receive radio frequencies; (c) *Acoustic systems*, which create and receive sound waves or reflected sound waves; (d) *Vision systems* that use some sort of laser ranging or cameras. A combination of some of the previous systems is considered as a *Hybrid system* [5]. A complete review of the different techniques available in the literature can be found in [6] and in the references in there. Among them, the most common systems are inertial and radio frequency. Within the radio frequency, the WiFi signal is the most frequently deployed in most of the indoor places for providing internet access, although there are other radio frequency proposals that use UWB (ultra wideband) systems, especially in industrial scenarios [1,7]. The ability to accurately determine the location of a device or individual indoors has gained significant attention in recent years, driven by the increasing demand for location-based services and applications. Traditional GNSS (global navigation satellite systems), such as GPS, while effective outdoors, suffer from signal attenuation and multipath effects in indoor environments, limiting their applicability. Wi-Fi, on the other hand, offers a promising alternative due to its widespread deployment and availability of infrastructure [8]. Basically, there are four main options for estimating the position using WiFi signals, which are based on, namely, RSS (received signal strength), ToF (time of flight), AoA (angle of arrival), or fingerprinting techniques. One of the most common methods is RSS-based localization [9,10], which estimates the distance between a device and an access point by using signal attenuation models; however, RSS is often subject to noise and environmental variability; what compromises the robustness and usability is complex and time-variant scenarios. Time of flight [11] is another method that measures the time it takes for a signal to travel from the access point to the device, providing a more accurate distance estimation in general but requiring precise timing synchronization, which is somehow difficult in time-variant and dynamic scenarios. Angle of arrival estimates the direction from which the signal reaches the device, using multiple antennas to triangulate the position [12]. Those techniques also suffer from complex and multi-path scenarios. More advanced techniques combine these methods or leverage fingerprinting where a pre-constructed map of RSS measurements across an area is compared to real-time data to estimate the device’s location [13]. These techniques, which are often used in tandem with ML (machine learning) algorithms, enhance the accuracy and robustness of WiFi-based localization [14,15]. There is a large variety of machine learning techniques for tackling this estimation problem. In this paper, a diverse set of ML algorithms for indoor localization have been evaluated, which encompass a range of methodologies and complexities. The selected algorithms include the following:

- Classification: KNN (K-Nearest neighbors), SVM (support vector machine), SVC (support vector classifier), RF (random forest), XGBoost, DT (decision trees), CatBoost, and NN (neural networks).
- Regression: CatBoost, KNN, RF, XGBoost, Lasso, Ridge, and Polynomial Regression.

This comprehensive suite of algorithms enables a thorough comparison of their performance in both building/floor classification and coordinate regression tasks. The main idea is to divide the estimation of the device’s position task into several subtasks of classification and regression, which are developed in steps.

KNN, a foundational supervised learning algorithm, is known for its simplicity and adaptability [16]. SVM and SVC have demonstrated effectiveness in classification problems, particularly in building and floor identification [17]. Ensemble methods such as Random Forest and XGBoost excel at improving prediction accuracy and robustness through decision tree aggregation [16]. CatBoost has garnered attention for its high performance in object detection tasks [18].

Thus, the localization problem is approached in a two-step process:

- Building and Floor Classification: This task utilizes classification algorithms to determine the building and floor corresponding to a given Wi-Fi fingerprint. Performance metrics include accuracy, precision, recall, and F1-score.
- Coordinate Regression: Once the building and floor are identified, regression algorithms are employed to estimate the longitude and latitude coordinates.

MSE (mean squared error) and MAE (mean absolute error) are used to evaluate regression performance. To optimize algorithm performance, hyperparameter tuning is conducted using *GridSearchCV* with cross-validation. This process helps to prevent overfitting and enhance generalization capabilities.

The UJIIndoorLoc dataset [19] serves as the experimental base for this study, providing a rich collection of Wi-Fi fingerprint data from three buildings with multiple floors. The dataset encompasses 20,000 samples collected from 25 Android devices, with 520 access points contributing to the fingerprint information.

In [20], the authors compare traditional techniques such as RSS-based, ToA, or fingerprint for indoor positioning, including a few ML techniques, solely focusing on signal strengths and physical measurement rather than the data-driven approaches here. The performance in there is different from the one presented here.

Additionally, the authors in the paper [21] proposed a solution BoF (bag of features) for indoor positioning based on fingerprints and machine learning. However, their proposal only uses the KNN technique and a few others in a simpler scenario. Moreover, it presents the localization problem as a whole instead of dividing it into different tasks. The MAE (unique performance measured in the paper) obtained in the experimental experiment is similar, although for a much smaller scenario with a lower density of WAP (20 times smaller and 14 times less dense deployment), and moreover, with a single floor.

Finally, a reduced experiment with the same dataset has been carried out in [22] but utilizing ANN (artificial neural network) with only one of the buildings and one of the floors. That is, they only used the dataset in one of the buildings and one of the floors to create an ANN model for this specific scenario. Their results showed 19.78 RMSE and 14.63 MAE, which are much larger than in our proposal. Moreover, our results are for a model that includes the whole scenario: three buildings with up to five floors.

Moreover, no optimization techniques were used in those research and experiments.

In this research, which differs from previous research, the authors aim to implement and evaluate multiple machine learning algorithms to comprehensively assess their performance in large-scale indoor localization. Furthermore, leveraging multiple access points enhances position triangulation, resulting in higher accuracy and reduced localization errors. Additionally, a novel architecture dividing the problem into different tasks is proposed.

The contributions and novelties of this paper are the following:

- A comprehensive evaluation of several ML techniques for the task of classification of the building and the floor and for estimating the latitude and longitude. To the best of the authors’ knowledge, there is no other research that makes a comparison of most machine learning techniques for the task of building and floor classification and for the coordinate regression under the same scenario.
- The use of a complex scenario with several buildings and floors for the estimation of latitude, longitude, and height (floor level), which tests the different techniques in a variety of tasks using the same set of parameters.
- The proposal of a three-step procedure to be used for this purpose.
- The evaluation of the *GridSearchCV* technique to optimize the hyperparameters and boost the global performance. The evaluation of this technique for indoor positioning classification in realistic scenarios has not been tested before.
- The identification of CatBoost and Random Forest as the most effective methods, achieving high accuracy in classification, is important and useful for further research and implementations. This empirical focus provides a clear advancement over traditional methods. The existing papers, while outlining the strengths and weaknesses of various techniques, do not provide direct comparisons of accuracy or results from empirical studies, which could limit the applicability of their findings.

The remainder of this paper is structured as follows: Section 2 provides an in-depth description of the scenario from which the dataset was obtained, highlighting its key characteristics. In Section 3, we offer a comprehensive review of various machine learning techniques applicable to these tasks. Following this, Section 4 details the proposed estimator architecture and Section 5 presents a discussion of the results. Finally, Section 6 concludes the paper by summarizing the key findings and insights.

## 2. Scenario

**Figure 1:**
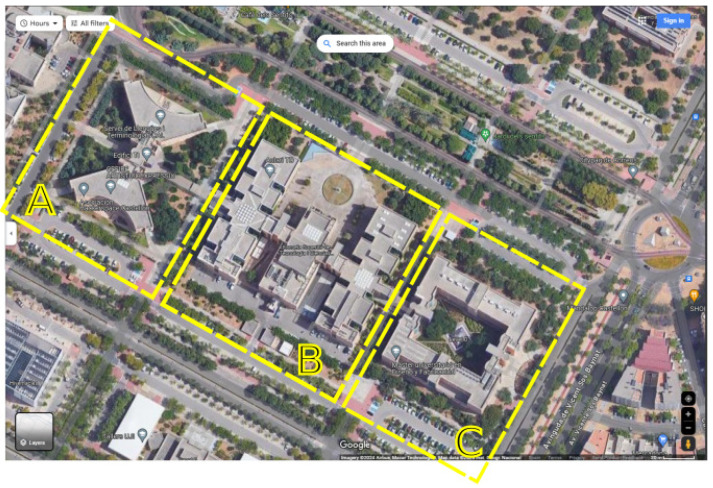
Top-view of the Technical departments of Jaime I university. Three buildings of the scenario. Includes three separated buildings. Building A: Informatics and Math departments. Building B: Teaching modulus. Building C: Sciences and technology.

In this paper, the *UJIIndoorLoc* dataset [19] is used. The scenario is an university campus with three buildings corresponding to the ESTCE (technical school for technology and experimental sciences, in Spanish) as shown in Figure 1. Those three buildings are close to each other and have some open areas (outdoor) where the WiFi signal is present and can be used for localization. Building A is for the Informatics and Math departments, building B is for teaching basically, and building C is for sciences and technology. The total area of the scenario is approximately 300 m × 100 m × 20 m (length × width × height).

Building A has a special shape of two opposite triangles and makes more difficult the uniform distribution of access points. There are four floors in this building. In this premises, offices, classes, and laboratories are mainly distributed symmetrically in both triangles. Since this configuration is the more challenging, most of the experiments have been shown with it. Building B is composed of four floors (one of them, floor number 1 is underground) and three parts, but the database does not show this distribution. Visually, building B is divided into three independent moduli that are connected through corridors as seen in the figure, but the dataset does not distinguish between the three different moduli, i.e., for the database, all the points belong to building B. The distribution of rooms and offices is more or less homogeneous in the three modulus of the building. Lastly, edifice C has five floors (one of them, floor 1, is underground), and the distribution of access points is around the perimeter of the square because of the distribution of the building.

The dataset includes 20,000 samples collected from three buildings with four or five floors on the Technical School at the Jaime I university campus. There are signals for 20 users with 25 Android devices. The devices can be inside/outside/in front of (at the door of an office, a corridor, or a classroom, showing the variety of places and measurements included in this dataset. The number of WAP (wireless access points) is 520, and since the *UJIIndoorLoc* dataset employs WLAN fingerprint positioning technologies, the signal strength of each building, floor, geographical coordinates, and access point is one of the prominent features of this dataset. The latitude and longitude in the dataset use pseudo Mercator EPSG:3857 units, so the coordinates are in meters instead of degrees.

The goal is to estimate the exact position—latitude, longitude, building ID, and floor—for any device only taking into consideration the RSSI (received signal strength indicator) from all the wireless access points. Of course, depending on the position of the device, not all the WAP will be received but only the closed ones.

The idea is based on the divide-and-conquer methodology, this is, split the estimation of the three coordinates—latitude, longitude and height—in three steps, as will be explained in the following sections.

Although this is a concrete scenario with the specific dataset, it is generic enough that the approach used in this paper can be easily extended to other similar or more complex scenarios. The main advantage of our proposal is that instead of attacking the estimation of the coordination problem as a whole in a complex scenario like this one with different buildings and floors, the approach reduces the problem each time to a more concrete task, either a classification or a regression. Those specific tasks are evaluated and compared with different ML techniques, and the best ones are selected. This knowledge is also very useful for future designs. Finally, the estimation of latitude and longitude is obtained by using a regressor model trained for specific buildings and floors.

## 3. Alternative Learning Algorithms

Once the example scenario has been described and the main idea has been introduced, the evaluation of different ML techniques is evaluated in this section for the different tasks in order to check robustness and accuracy.

### 3.1. Classification

As indicated, the task of positioning is split into two parts: the classification of the building and floor and the estimation of the latitude and longitude as a regression.

Let us start with the classification techniques. KNN (K-nearest neighbor) is one of the simplest and most effective algorithms for classification and regression tasks, often applied in object detection scenarios. KNN is a non-parametric, supervised learning algorithm that classifies or predicts the value of new data points based on the *K*-nearest training samples in the feature space. By selecting *K* nearest neighbors and evaluating the majority class (for classification) or the average value (for regression), KNN assigns new data points to the appropriate class or predicts their value. Despite its simplicity, KNN faces challenges in high-dimensional spaces, particularly in terms of computational cost and distance metric sensitivity. These issues can be alleviated by optimizing the parameter *K* and using appropriate distance metrics [16].

SVM (support vector machines) have been widely used in both classification and regression tasks due to their ability to create robust decision boundaries. SVM works by finding the optimal hyperplane that maximally separates the data points belonging to different classes in the feature space. When dealing with non-linear data, SVM employs kernel functions (e.g., polynomial, Gaussian, or radial basis functions) to project data into higher-dimensional spaces where linear separation becomes feasible. The algorithm excels in scenarios with small datasets and a clear margin of separation, although it may struggle with highly noisy data or overlapping classes. The kernel trick enables SVM to handle complex and non-linearly separable data efficiently, provided that Mercer’s condition is met [23].

DT (decision trees) are a highly interpretable model used for both classification and regression tasks. The model splits the database at the most discriminative feature at each node, leading to the creation of branches and leaves where the prediction is made. Decision Trees are easy to understand, transparent, and computationally efficient. However, they are prone to overfitting, particularly when the tree grows too deep. Pruning techniques and the use of ensemble methods, such as Random Forest, can mitigate this issue, making DT a powerful tool in practical applications [24].

RF (random forest) is a versatile ensemble learning method widely used for both classification and regression tasks. It is a tree-based model that builds multiple decision trees during training and outputs the class that is the mode of the classes (classification) or the mean prediction (regression) of the individual trees. The model introduces randomness by selecting random subsets of features and data points, thereby reducing overfitting and improving generalization. Random forest is particularly effective when dealing with large datasets and noisy data, offering superior performance due to its ensemble approach. The model’s recursive splitting of the dataset ensures that the classification is performed in an iterative manner until the stopping criterion (e.g., the minimum number of samples per node) is met [25].

XGBoost is a scalable and efficient gradient boosting algorithm specifically designed for large-scale data mining tasks. It builds trees sequentially, where each tree attempts to correct the errors of the previous trees. XGBoost optimizes the loss function using a second-order Taylor expansion, which provides both gradient and Hessian information, improving the convergence speed. This algorithm is particularly effective in handling sparse data, and it includes built-in mechanisms for regularization, reducing the risk of overfitting. Moreover, XGBoost’s support for GPU (graphics processing units) acceleration allows it to handle large datasets efficiently, making it up to ten times faster than traditional gradient boosting algorithms [26].

NN (neural networks) are a class of deep learning models inspired by the structure of the human brain, consisting of interconnected neurons organized in layers. NN models have shown remarkable performance in tasks such as object detection, especially when large and complex datasets are involved. NNs are capable of learning high-level features from raw data, making them suitable for a wide variety of tasks, including image classification, speech recognition, and more. The architecture of an NN can vary significantly, from simple feedforward networks to deep convolutional and recurrent networks, allowing flexibility in addressing different problem domains [27].

CatBoost is a state-of-the-art gradient boosting algorithm that performs exceptionally well in handling categorical data without the need for extensive pre-processing. This algorithm leverages both CPU and GPU for efficient computation, allowing it to outperform other gradient boosting libraries such as XGBoost and LightGBM. CatBoost’s ability to manage high-cardinality categorical features makes it particularly well-suited for classification tasks, while its built-in overfitting prevention mechanisms ensure robust generalization across various datasets [28].

### 3.2. Regression Models

Four distinct regression models were employed in this study to address specific regression tasks besides the previous techniques that were valid either for classification and regression: LASSO, Ridge, and Polynomial Regression.

LASSO (Least absolute shrinkage and selection operator) performs feature selection by forcing the coefficients of insignificant features to zero, resulting in a simpler model that improves interpretability and reduces overfitting. The regularization parameter controls the degree of shrinkage, with larger values leading to more aggressive feature elimination [29].

Ridge Regression addresses multicollinearity issues by imposing an L2 penalty on the size of coefficients. Unlike LASSO, Ridge does not reduce coefficients to zero but instead shrinks them, thus reducing their impact on the prediction. This makes Ridge more suitable when many features are relevant but only weakly correlated with the target variable.

Polynomial Regression extends linear models by considering polynomial terms of the independent variables. This allows the model to capture non-linear relationships between the predictors and the target variable, increasing flexibility at the cost of introducing more complexity into the model [30].

## 4. Proposed Architecture

In the previous section, the different ML techniques that were going to be checked were described. In this section, the proposal will be described and analyzed.

**Figure 2:**
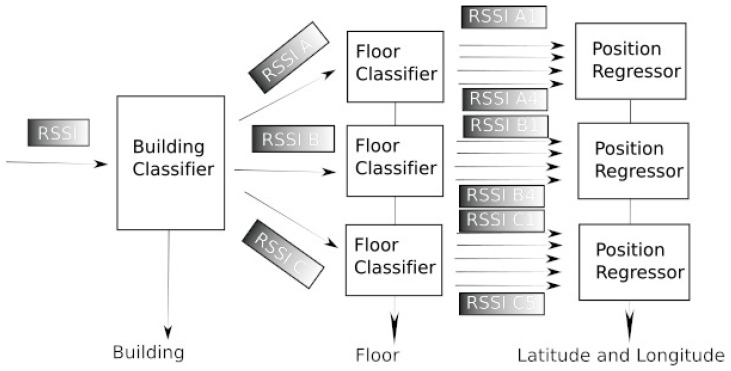
Proposed Architecture block diagram. The different subsets of RSSI parameters are specified. Buildings A and B have 4 floors, while building C has 5 floors.

As it has indicated earlier, the idea is based on divide-and-conquer. In our case we split the original problem of estimating the latitude, the longitude, and the height, which is complex if we take it as a whole and divide it into a three-step estimation. In fact, the first step and the second are converted into a classification problem and the last one into a regression task. In this way, we can tackle classification techniques easier while using a subset of all the measurements, simplifying the problem and making it more robust. Moreover, instead of using all the variables (measurements) for the regression problem of coordinates, only the more relevant ones are used for the last step of regression. It is well known that the high dimension of data can be beneficial for classification problems, but it usually generates noise in regression algorithms. With our approach, the regression is carried out with a reduced number of variables. Thus, our proposal is as follows. The first step involves all the RSSI measurements from all the WAP, including those that their value is 0 because the terminal is far away from that WAP and it does not receive a signal from it (in the dataset, this value is fixed to 100). In the UJIIndoorLoc dataset, this is a vector of 520 values for each device (one value for each WAP). With those measurements, we design a building classifier that identifies the building where the terminal is. After the building has been identified, since there are three buildings, A, B, and C, three floor classifiers are designed, one per building. The main subset of values from original RSSI will be identified to reduce the model’s complexity, and thus, the input of these classifiers, instead of being RSSI, it will be a subset of it, denoted as $RSSI_{A}$, $RSSI_{B}$ and $RSSI_{C}$ for building A, B, and C, respectively. In this way, the variables used in the model for building A, for example, will likely be the measurements from WAP located in building A (remember that the position for WAP is unknown). Finally, once the building and the floor have been obtained, different regressors are designed for each floor to obtain the latitude and longitude. It is easy to observe that the input for those regressors will be a subset of RSSI, denoted as $RSSI_{x,y}$, where *x* is either A, B, or C, and *y* is a number from 1 to 5 (since there are up to five floors in some of the buildings). This way, a sort of transfer learning scheme is designed, because at each step and model, some previous knowledge (learning) is incorporated. The architecture of the proposed estimator is depicted in Figure 2, and it can be easily extended with more buildings and/or floors, or a more complex scenario.

Moreover, the reduction in matching and operations for real-time calculation volume lies in this hierarchical structure. By first classifying high-level features (i.e., building and floor), we can significantly reduce the search space for finer-grained fingerprint matching (RSSI values in the subsequent subset such as $RSSI_{A}$ or $RSSI_{x,y}$ for example), which would otherwise require a much larger and computationally intensive comparison across all potential locations. As a drawback, this architecture needs the training of several models for building identification, floor classification, and latitude and longitude estimation.

## 5. Discussion

After describing the proposed architecture for estimation, different ML techniques are evaluated to decide which ones are the best candidates for each of the steps.

### 5.1. Performance Metrics

First of all, the three main criteria usually used in evaluating ML techniques are summarized in the following. Evaluating the performance of machine learning models is crucial to ensure their reliability and effectiveness. Several metrics are commonly used, each highlighting different aspects of the model’s performance. Here, key evaluation metrics such as *Precision*, *Recall*, and *F1-Score* are described.

Precision is the ratio of correctly predicted positive observations to the total predicted positives. It is useful when the cost of false positives is high. Mathematically, precision is defined as

(1) $$\mathrm{Precision}=\frac{TP}{TP+FP}$$

where

- TP is the number of true positives.
- FP is the number of false positives.

A high precision score indicates a low false positive rate.

On the other hand, *Recall*, also known as *Sensitivity* or *True Positive Rate*, is the ratio of correctly predicted positive observations to all actual positives. It is particularly important when the cost of false negatives is high. The recall is calculated as

(2) $$\mathrm{Recall}=\frac{TP}{TP+FN}$$

where FN is the number of false negatives. A high recall indicates that most positive examples are correctly identified. Then, the *F1-Score* is the harmonic mean of precision and recall, providing a balance between the two. It is especially useful when the class distribution is imbalanced. The formula for the F1-Score is given by

(3) $$F1=2\cdot \frac{\mathrm{Precision}\cdot \mathrm{Recall}}{\mathrm{Precision}+\mathrm{Recall}}$$

This metric is a compromise between precision and recall and is optimal when both are equally important.

Another important metric is the *Accuracy*, which is the ratio of correctly predicted observations to the total observations. It is a commonly used metric, but it may be misleading if the classes are imbalanced. The formula is

(4) $$\mathrm{Accuracy}=\frac{TP+TN}{TP+TN+FP+FN}$$

where

- TN is the number of true negatives.
- FN is the number of false negatives.

### 5.2. Building Classification

**Figure 3:**
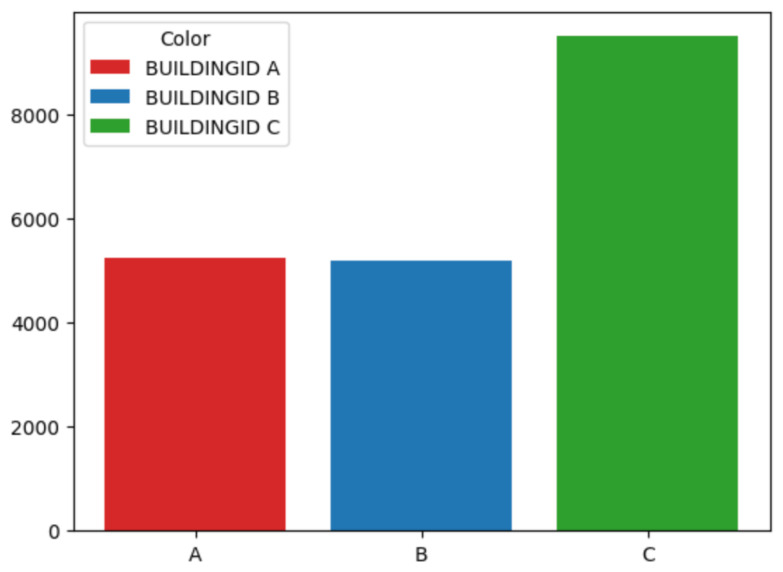
Building Count. Number of samples per building.

For the classifier in charge of identifying the building, several ML techniques have been checked. First of all, the number of samples per building has been normalized because, as can be seen in Figure 3, there were almost twice as many samples for building C as the other two. The whole dataset has been used for training and testing to distinguish the intended building in which the object is located.

**Figure 4:**
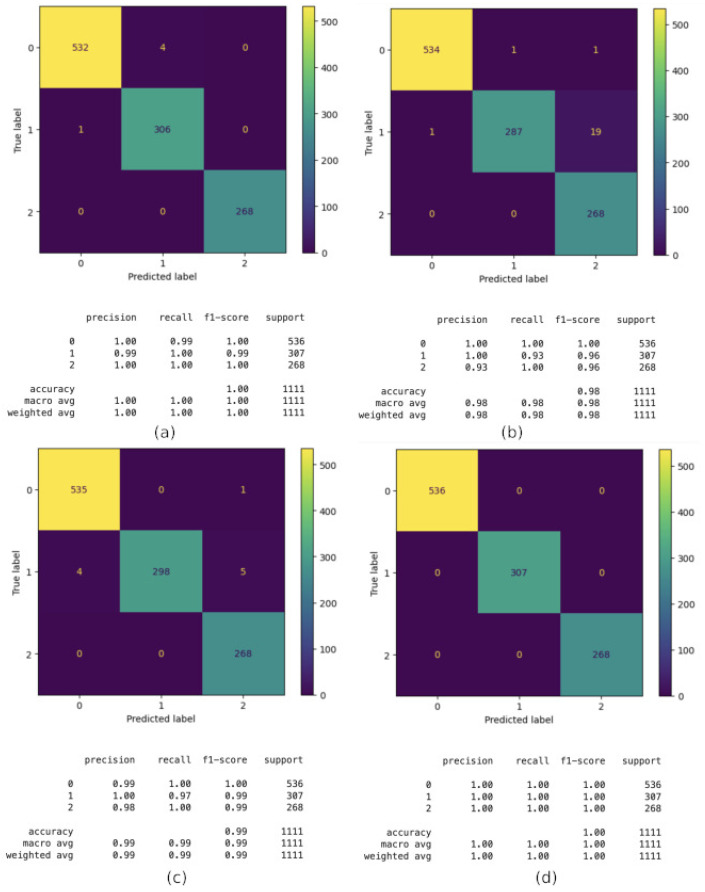
Performance of different techniques in the building classification. (**a**) CatBoost; (**b**) Decision Tree; (**c**) KNN; (**d**) Random Forest.

The following techniques have been evaluated, namely, CatBoost, Decision Tree, KNN, and Random Forest. The evaluation is according to a number of criteria, including recall, precision, F1 score, and accuracy, as previously described. As can be seen in Figure 4, all the classifiers obtain a good performance in identifying the building with accuracy near 100%. Moreover, CatBoost and Random Forest obtained a perfect classification, and they will be the algorithms used in our final proposal for the first step.In this figure, the confusion matrices, along with the main parameters, are shown.

### 5.3. Floor Classifiers

**Figure 5:**
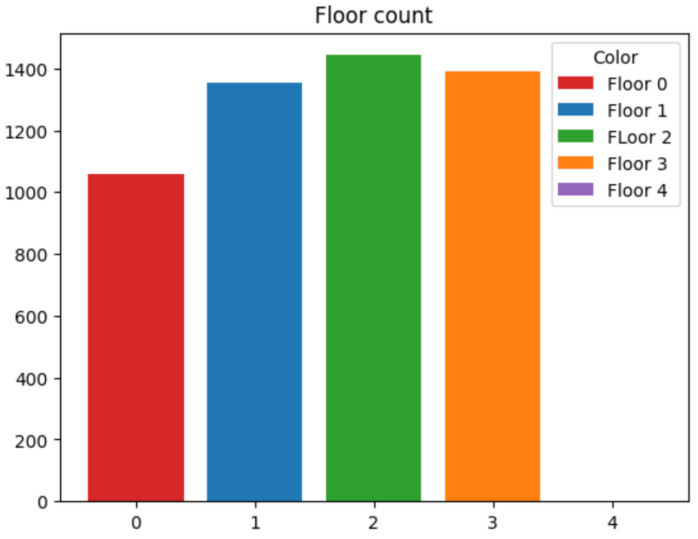
Floor Count samples at building A. This building only has 4 floors.

After the building is identified, the next step is to classify the floor that the device is on. For this purpose, a different subset of input data, based on the building ID are used. Several classification techniques have also been evaluated. As in the previous case, analyzing the number of samples for each floor, the number is non homogeneous (as it can be observed in Figure 5 for building A), and thus, some regularization should be approached to guarantee a fair and efficient training process.

Similar results have been obtained for the three buildings, and thus, without loosing generalization and for shortening the results, the performance for building A is shown in detail in the following. Moreover, the results for building A were the worst ones because the architecture of this building was more challenging for the algorithms (remember that building A was two triangles joined by their vertex).

**Figure 6:**
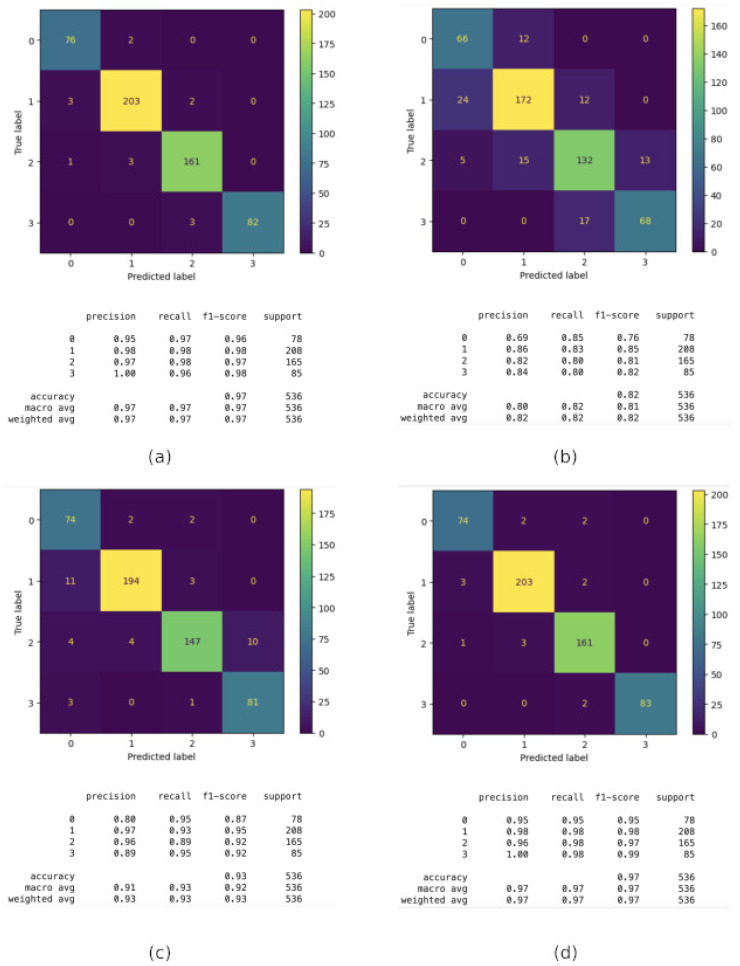
Floor classification performance comparison for Building A: (**a**) CatBoost; (**b**) Decision Tree; (**c**) KNN; (**d**) Random Forest.

**Figure 7:**
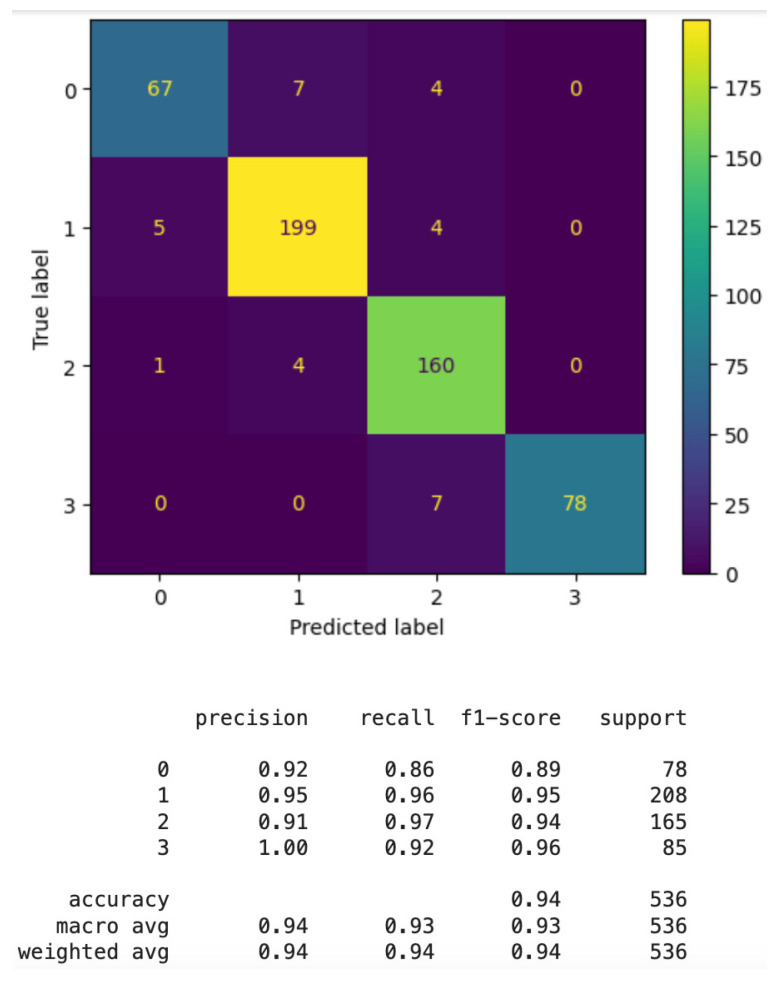
Floor classification performance in Building A for Support Vector Machine.

As in the building classification, the same techniques have been studied but however, SVM have also been incorporated to the analysis. As it can be observed in Figure 6 and Figure 7, again, CatBoost and Random Forest provide the best performance and accuracy of 97%. Interestingly, for all the techniques, the performance for floor 2 and 4 (1 and 3 in the figures) are much better than for floor 1 and 3 (0 and 2 in the figures). The explanation could be the distribution of the WAP in these floors and the topology of the building with more classes than in the other two floors for building A.

In summary, CatBoost and Random Forest are the top-performing models, both achieving an accuracy of 97%. These models excel in handling the classification task with very few misclassifications. The SVM model follows closely with an accuracy of 94%. It is a reliable model but slightly less effective than CatBoost and Random Forest, so we can conclude that is a moderate performer. then, KNN and Decision Tree models show lower performance, with Decision Tree being the least accurate at 82%. These models struggle more with classifying certain instances, leading to higher misclassification rates.

Similar results have been obtained for buildings B and C. Those results are not included to avoid the repetition of similar figures.

In order to improve even more the performance, an optimization of the hyperparameters has been carried out.

#### GridSearchCV

In the field of machine learning, hyperparameter tuning is crucial for improving model performance. *GridSearchCV*, part of the scikit-learn library is a widely used technique for performing an exhaustive search over a specified parameter grid to identify the optimal hyperparameters for a given estimator. This process aims to maximize the model’s predictive accuracy by systematically evaluating combinations of hyperparameters.

*GridSearchCV* automates the process of hyperparameter optimization by conducting a brute-force search across a defined parameter space. Hyperparameters differ from learned parameters in that they are external to the model and cannot be estimated from the data during training. Examples include the learning rate in gradient descent or the number of trees in a random forest. Manually choosing these values is often infeasible, as models can contain multiple hyperparameters, each taking on numerous possible values.

The parameter grid is a dictionary that maps model parameters to lists of values to explore. For example, when tuning an SVM, the parameter grid might include various values of regularization (L) and kernel types.

The *cross-validation* technique, denoted as *k*-fold cross-validation, divides the dataset into *k* equally sized folds. For each hyperparameter combination, the model is trained on k−1 folds and validated on the remaining fold. This process is repeated *k* times, with a different fold serving as the validation set each time. The average performance metric across all folds determines the effectiveness of the hyperparameter set.

The loss function *L* is computed as:

(5) $$L=\frac{1}{k}\sum_{i=1}^{k}L\left(y_{\mathrm{true}}^{\left(i\right)},y_{\mathrm{pred}}^{\left(i\right)}\right)$$

where L is a loss function (e.g., MSE for regression or log-loss for classification), $y_{\mathrm{true}}^{\left(i\right)}$ represents the true labels, and $y_{\mathrm{pred}}^{\left(i\right)}$ represents the predicted labels for the *i*-th fold.

Upon completion of the search, *GridSearchCV* provides the optimal hyperparameters that yield the best average performance across all folds. Additionally, it returns the estimator refit on the entire training set using these best parameters. This ensures that the model benefits from the full dataset while leveraging the best hyperparameter settings discovered during cross-validation.

Not all are advantages in this technique, as it can be imagine, one of the limitations of *GridSearchCV* is its computational expense. The complexity increases exponentially with the number of hyperparameters and their values. If there are *p* hyperparameters, each with $n_{i}$ possible values, the total number of models evaluated is:

(6) $$\prod_{i=1}^{p}n_{i}\;.$$

For high-dimensional or large datasets, this can become computationally prohibitive. In such cases, alternative techniques like *RandomizedSearchCV*, which samples a fixed number of random combinations from the grid, can be more efficient.

Although during *GirdSearchCV* process the dataset is recombined, it is worth noting that once the hyperparameter has been optimized, the training process is carried out as usual with the original dataset (training and validation) but with tuned hyperparameters, so that consistency is not compromised.

**Figure 8:**
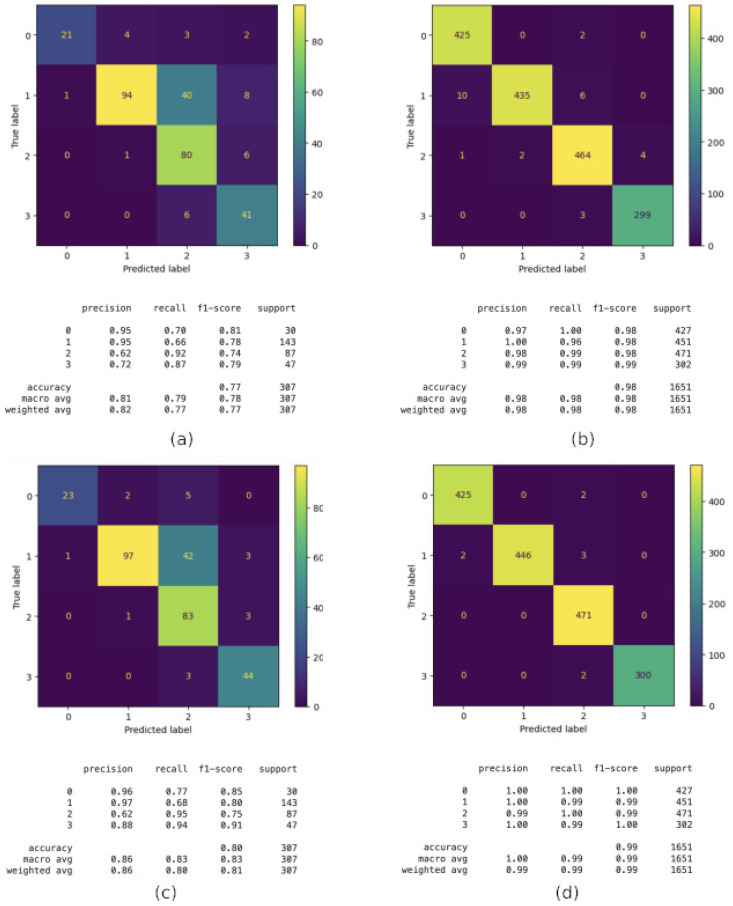
Floor classification enhancement comparison by using GridSearchCV: (**a**) CatBoost; (**b**) CatBoost + SG optimization; (**c**) Random Forest; (**d**) Random Forest + SG optimization.

Thus, for the best algorithms, we have enhanced performance by using GS (GridSearchCV) to optimize the hyperparameters. This is, *GridSearchCV* has been applied to CatBoost and Random Forest to tune their hyperparameters. The results of this enhancement are shown in Figure 8. The number of samples in GridSearchCV exceeds that of the original dataset due to the cross-validation method employed. Initially, the dataset is divided into 70% for training and 30% for testing. Within *GridSearchCV*, the 70% training portion is further divided into 5 subsets. The training and testing processes are then conducted five times, each time using one subset for testing while the remaining four are used for training. This approach is designed to identify the optimal hyperparameters. Once these hyperparameters are established, the model is evaluated on the remaining 30% of the dataset to assess its performance.

For example, the 70% training set is divided into five subsets: during the first iteration, the first subset is used exclusively for testing, while the others are used for training. In the second iteration, the second subset is used for testing, with the remaining subsets used for training, and so on.

As can be seen in this figure, the small problems in classifying floors 2 (1 in the image) and 3 (2 in the image) or 3 and 4 (2 and 3 in the image, respectively) using catBoost disappear after optimization with *GridSeaarchCV*. Similar behavior occurred with random forest and its optimized results.

**Figure 9:**
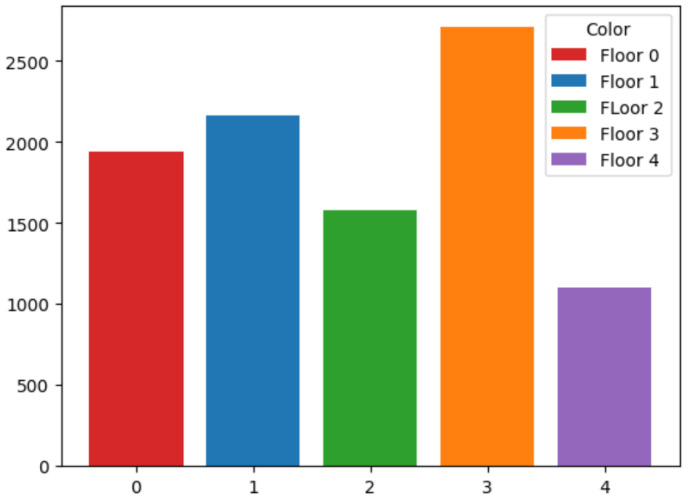
Floor Count.

On the other hand, building C has five floors instead of 4 as the other two buildings. In order to show the performance and for conciseness, only the performance obtained by the best techniques, i.e., CatBoost, and Random Forest, is shown for this building. In Figure 9, the distribution of samples for each of the floors is plotted. As in the other cases, a regularization to obtain the same number of samples for all the floors is carried out.

**Figure 10:**
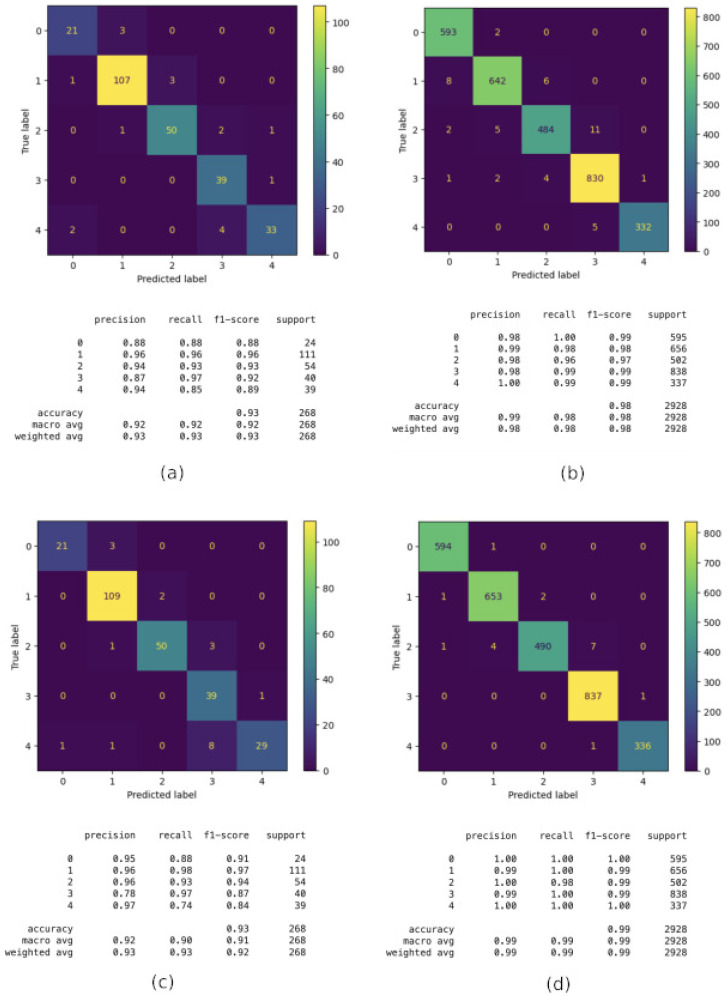
Floor classification performance in Building C: (**a**) CatBoost; (**b**) CatBoost after using GridSearchCV; (**c**) Random Forest and (**d**) Random Forest after using GridSearchCV.

In this building, the best models are still CatBoost and Random Forest, with 93% initial accuracy. However, after applying the *GridSearchCV* technique, their performance boosted up to 98% and 99%, respectively, as can be observed in Figure 10. Looking into more detail, CatBoost (Image a) obtains Accuracy: 0.93, Weighted Precision: 0.93, Weighted Recall: 0.93 and Weighted F1-Score: 0.93. This model shows good overall performance, with most classes being well classified, especially floor 4 (3 in the figure). However, there is some confusion when classifying floor 5 (4 in the image) that sometimes it is classified as floor 3 (4 in the image), suggesting the model struggles to differentiate between these two floors. After CatBoost is Optimized with GridSearchCV (Image b), the performance increases to Accuracy: 0.98, Weighted Precision: 0.98, Weighted Recall: 0.98 and Weighted F1-Score: 0.98. The optimization through *GridSearchCV* significantly improved the model’s performance. There is a noticeable increase in precision, recall, and F1-Score compared to the non-optimized model. Floors 1 (0 in the figure) and 4 (3 in the figure), which previously showed some confusion, are now almost perfectly classified, with very few incorrect predictions.

On the other hand, Random Forest (Image c) obtains Accuracy: 0.93, Weighted Precision: 0.93, Weighted Recall: 0.93 and Weighted F1-Score: 0.92. The Random Forest model performs similarly to the non-optimized CatBoost, with an accuracy of 0.93. However, it shows more confusion between floors 4 (3 in the figure) and 5 (4 in the figure) compared to CatBoost, reflected in a lower F1-Score for these floors. The model seems less effective at distinguishing between certain classes, particularly floor 5 (4 in the figure). Then, after it is Optimized with GridSearchCV (Image d), Random Forest obtains the following: Accuracy: 0.99, Weighted Precision: 0.99, Weighted Recall: 0.99, and Weighted F1-Score: 0.99. As with CatBoost, optimization with *GridSearchCV* significantly improves the Random Forest model’s performance. The metrics are very high, with an accuracy of 99%. Nearly all classes are perfectly classified, indicating that the optimized model is highly effective. We can conclude that optimization with GridSearchCV significantly improves the performance of both models. This is evident in the increase in all key metrics (accuracy, precision, recall, F1-Score). Moreover, before optimization, both models performed similarly. However, after optimization, both achieved near-perfect performance, with the optimized Random Forest model showing a slight edge in metrics, reaching 99% in precision, recall, and F1-Score. Both models, before optimization, show difficulty distinguishing between floors 4 and 5 (3 and 4 in the figure, respectively). However, optimization nearly eliminates this confusion.

In summary, both CatBoost and Random Forest are strong models for this classification task, but hyperparameter optimization is crucial for maximizing their performance. The Random Forest model optimized with *GridSearchCV* provides the best performance in this case, with near-perfect classification.

These two techniques allow a very high accuracy (nearly 100%) for the classification of the floor, which, when combined with the high accuracy in the building identification, provides the best conditions for the last step, the estimation of the latitude and longitude.

### 5.4. Latitude and Longitude Estimation

Finally, the last step is to estimate the latitude and longitude that, jointly with the floor, offers the precise localization envisaged. This is a regression problem, and several ML algorithms have been evaluated for this purpose, namely, RF, CatBoost, SVM, XGB, and NN. Those techniques have been applied to design the estimators for latitude and longitude for the devices on the different buildings and floors. Additionally, other techniques exclusively for regression have been evaluated, as they will be discussed later.

Since the results are similar in all the buildings, and for the sake of conciseness, in the following, only results for building A are shown on the following Table 1, Table 2, Table 3 and Table 4. In these tables, the best algorithms have been highlighted in bold and with green color for ease identification.

As it can be extracted from these tables, Random Forest Regressor, Catboost Regressor and XGBoost regressor are the three best techniques that obtain the best performance in terms of MAE and MSE. CatBoost exhibited, in general, a slightly better performance than RF and XGBoost.

In order to boost the performance, also *GridSearchCV* optimization has been applied to RF, CB and XGBoost. The results are highlighted in the tables. It can be easily observed that the performance is boosted significantly, especially in terms of MSE. Again, Catboost offers slightly better results.

In order to complete the analysis and for comparison purposes, also LASSO, Polynomial and Ridge have been used. In this case, the results are for building C and floor 1, and the summary is presented in Table 5. Those results show a very low performance and this is the reason why they have been discarded for further enhancements.

Also for completeness, and to explore the benefits of using *GridSearchCV* potential, it has been applied to all the algorithms. A summary for building C and floor 2 is shown in Table 6.

In general, CatBoost with Grid Search (CB + GS) consistently outperformed other models across all floors and buildings. It provided the lowest errors in both MAE and MSE for latitude and longitude estimation. Moreover, Random Forest with Grid Search (RF + GS) and XGBoost with Grid Search (XGB + GS) were also strong performers, often following closely behind CB + GS, highlighting the effectiveness of ensemble methods combined with hyperparameter optimization. On the other hand, SVM, even with Grid Search, showed the worst performance overall, suggesting it is not suitable for this specific application of indoor WiFi localization. And NN showed moderate performance but were generally outperformed by ensemble methods with optimized hyperparameters.

*GridSearchCV* consistently improved the performance of all models tested, demonstrating the importance of hyperparameter tuning in machine learning applications.

The authors in the paper [21] proposed a solution BoF for indoor positioning based on fingerprints and machine learning, however, their proposal only uses KNN technique into a simpler scenario. Moreover, it presents the localization problem as a whole instead of dividing it into different tasks. The obtained MAE in the experimental experiment is around 1.6 m for a scenario with five wireless access points in 15 m × 11 m (a small apartment with only one floor), which is similar to our findings but in a scenario 20 times larger. In fact, the MAE they obtained for a simulated scenario of 50 m × 50 m was around 2.8 m. much larger than our proposal. It should be noted that the density of WAP in our scenario is around 1154 m^3^ per WAP (300 m × 100 m × 20 m = 600,000 m^3^/520 WAP) while in [21], the density is 82.5 m^3^ per WAP (15 m × 11 m × 2.5 m = 412.5/5 WAP). Indeed, in [21], when they increased the scenario in their simulations, the MAE increased significantly. Moreover, our experiments are with up to 5 floors, while the former was on a single floor.

Finally, the reduced experiment with the same dataset in [22] using ANN but for a single building and single floor obtained an MAE of 14.63 m, which is much larger than our proposal.

### 5.5. Final Design

Thus, the final design of the system can be developed by using CatBoost techniques for building a classifier, floor identification, and latitude and longitude estimation. Another alternative is to replace CatBoost by Random Forest techniques for those tasks. Both designs will lead to similar global performance.Alternatively, the last step (longitude and latitude estimation) can be carried out also by XGBoost, obtaining similar results as in the other cases. This is, the final design can be built using CatBoost or Random Forest algorithms. This is interesting and very useful in the design and implementation of the indoor location because it provides flexibility in the design. Although CatBoost is more complex than Random Forest because the decision trees need to be sequentially trained, it usually gives better performance in more complex scenarios, and if you do not have a large collection of data for training. Thus, for scenarios where the number of buildings, floors, and configurations of WAP is large, this can be the solution. On the other hand, Random Forest is simpler than Cat Boost because decision trees can be trained in parallel, so they are efficient for large volumes of data. However, their performance is slightly worse than CatBoost. Thus, in more simple scenarios, without doubt, their use would be more convenient.

Although these results are valid for the specific scenario in the UJIIndoorLoc dataset, the architecture can be easily extended to other scenarios. Moreover, the analysis of the techniques and the selected ones are useful for future designs and different scenarios.

## 6. Conclusions

In this paper, various machine learning techniques have been employed to accurately estimate latitude, longitude, and height (floor level) in indoor environments. The proposed approach is a three-stage estimator. First, it identifies the building where the device is located with an extremely high accuracy of 100%. Next, it determines the floor on which the device is situated, achieving 99% accuracy. Finally, it estimates the longitude and latitude with a high degree of precision.

A range of machine learning techniques was tested to identify the most effective ones. The key findings indicate that CatBoost and Random Forest are the most suitable methods for the classification tasks relating to building and floor identification. For the regression tasks of estimating latitude and longitude, CatBoost, Random Forest, and XGBoost were found to be the best options.

The role of hyperparameters has proven to be critical in this context. Consequently, a specific optimization technique, *GridSearchCV*, was utilized to fine-tune the hyperparameters, resulting in a significant performance improvement from approximately 90% to nearly 99%.

Although the model was applied to a scenario involving three buildings with several floors (up to five), the methodology is easily extendable to more complex and varied scenarios, yielding similar results. 

## Figures and Tables

**Table 1:** Building A/Floor 1 Performance Analysis.

F.	MAE/MSE	RF	RF + GS	CB	CB + GS	SVM	XGB	XGB + GS	NN
1	MAE.long.	5.53	**1.74**	5.95	**1.72**	15.45	6.24	**1.75**	8.50
	MAE.lat.	5.11	**1.23**	4.46	**1.52**	12.58	5.35	**1.44**	7.19
	MSE.long.	92.49	**13.32**	110.08	**9.51**	441.33	111.52	**15.53**	121.90
	MSE.lat.	83.06	**4.27**	43.53	**5.00**	235.16	108.96	**6.79**	89.71

**Table 2:** Building A/Floor 2 Performance Analysis.

F.	MAE/MSE	RF	RF + GS	CB	CB + GS	SVM	XGB	XGB + GS	NN
2	MAE.long.	5.18	**2.11**	5.50	**2.13**	18.07	5.14	**2.21**	8.18
	MAE.lat.	6.56	**1.63**	5.54	**1.65**	12.50	6.53	**1.64**	8.94
	MSE.long.	83.50	**17.11**	76.88	**14.69**	512.42	82.83	**23.06**	127.90
	MSE.lat.	128.66	**9.87**	71.83	**7.16**	230.42	163.58	**10.35**	145.160

**Table 3:** Building A/Floor 3 Performance Analysis.

F.	MAE/MSE	RF	RF + GS	CB	CB + GS	SVM	XGB	XGB + GS	NN
3	MAE.long.	4.43	**2.05**	4.67	**1.97**	11.74	4.83	**1.82**	5.50
	MAE.lat.	3.78	**1.75**	3.93	**1.86**	13.42	4.64	**1.80**	8.08
	MSE.long.	48.36	**11.38**	40.79	**10.46**	280.53	58.08	**12.82**	51.77
	MSE.lat.	33.45	**10.45**	30.53	**8.53**	251	50.61	**12.13**	113.03

**Table 4:** Building A/Floor 4 Performance Analysis.

F.	MAE/MSE	RF	RF + GS	CB	CB + GS	SVM	XGB	XGB + GS	NN
4	MAE.long.	4.52	**1.64**	3.96	**1.56**	14.24	4.90	**1.70**	6.07
	MAE.lat.	4.55	**1.76**	4.90	**1.81**	14.13	5.20	**1.63**	7.71
	MSE.long.	45.85	**6.62**	30.24	**5.84**	381.38	46.23	**9.20**	70.78
	MSE.lat.	46.41	**7.71**	49.82	**8.41**	296.35	55.17	**10.65**	105.83

**Table 5:** Building C/Floor 1 Performance Analysis.

Floor	MAE/MSE	Polynomial	Ridge	Lasso
1	MAE.long.	1,760,921,736.30	132.47	127.26
	MAE.lat.	5,804,107,449.44	96.39	92.95
	MSE.long.	1.1562030535598625 × $10^{19}$	29,112.63	27,201.81
	MSE.lat.	1.256101956925712 × $10^{20}$	14,564.96	14,234.70

**Table 6:** Building C/Floor 2 Performance Analysis.

Floor	MAE/MSE	RF + GS	CB + GS	SVM + GS	XGB + GS	DT + GS
2	MAE.long.	**2.18**	**2.59**	20.50	**2.17**	4.14
	MAE.lat.	**2.02**	**2.24**	11.31	**1.97**	4.44
	MSE.long.	**16.44**	**17.40**	511.64	**16.48**	84.02
	MSE.lat.	**11.01**	**11.49**	174.42	**12.54**	80.10

## Data Availability

Data are contained within the article.

## Abbreviations

The following abbreviations are used in this manuscript:

AoA	Angle of arrival
DT	Decision tree
GNSS	Global navigation satellite system
GPU	Graphics processing unit
GPS	Global positioning system
GS	GridSearchCV technique
IoT	Internet of things
KNN	K-nearest neighbours
MAE	Mean absolute error
ML	Machine learning
MSE	Mean squared error
NN	Neural networks
RF	Random forest
RMSE	Root mean squared error
RSS	Received signal strength
RSSI	Received signal strength indicator
SVC	Support vector classifiers
SVM	Support vector machines
ToF	Time of flight
WAP	Wireless access point
